# Detection of alpha radionuclides in air from patients during Ra-223 alpha radionuclide therapy

**DOI:** 10.1038/s41598-018-29449-9

**Published:** 2018-07-20

**Authors:** Seiichi Yamamoto, Katsuhiko Kato, Naotoshi Fujita, Masato Yamashita, Takuya Nishimoto, Hiroshi Kameyama, Shinji Abe

**Affiliations:** 1 Radiological and Medical Laboratory Sciences, Nagoya University Graduate School of Medicine, Nagoya, Japan; 2 Department of Medical Technology, Nagoya University Hospital, Nagoya, Japan

## Abstract

Ra-223 has recently been introduced to alpha radionuclide therapy. According to the decay scheme of Ra-223, an inert gas, Rn-219 is released from patients during alpha radionuclide therapy and its daughter radionuclides may accumulate around the patient. However, the concentration of these radon daughters during alpha radionuclide therapy was not obvious. Here, we first detected the radon daughters of Rn-219 around patients during alpha radionuclide therapy. While the Ra-223-administered patients were in a room for ~1.5 hours, the radon daughter concentration increased to 4 to 5 times higher than without the patients. When the patients were in the room, the energy spectra of the alpha particles in the air showed the peak of the radon daughter of Rn-219, Bi-211 (6.6 MeV), which was different from that without the patients. We conclude that the daughter radionuclides of Rn-219 are accumulated around the patient, and the concentration was higher than that of the natural radon daughters. However, the increase in levels of alpha emitters, while detectable, is lower than the daily variations and thus is likely not a source of concern for radiation exposure.

## Introduction

Recently, alpha-emitting radionuclides have been clinically used for radionuclide therapy. Since alpha particles have an advantage over beta particles in their short range and high linear energy transfer (LET), effective treatment is expected in radionuclide therapy^1,2^. With these advantages of alpha radionuclide therapy, Ra-223 is starting to be used in hospitals^3^. It is used for treatment of castration-resistant prostate cancer that is metastatic to bone^1,4^.

**Figure 1:**
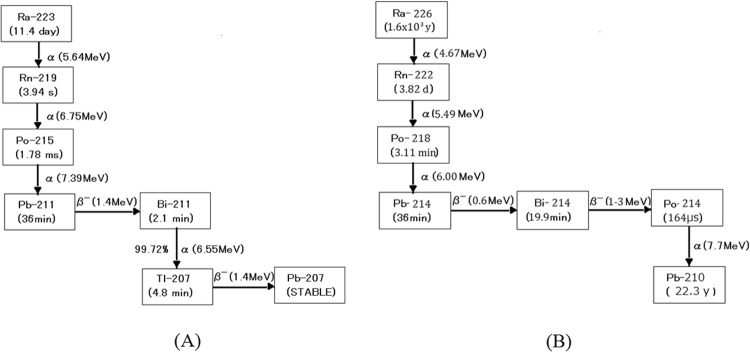
Major decay chains of Ra-223 (**A**) and Ra-226 (**B**).

According to the decay scheme of Ra-223, an inert gas, Rn-219 is released from the patients during alpha radionuclide therapy. We show the decay chain of Ra-223 in Fig. 1(A). Since the half-life of Rn-219 is short (3.94 s) and the decayed products are not a gas, it is thought that the escape of Rn-219 from the patient is very small. However, by the respiration of the patients, some of the Rn-219 in the body may escape from the patients and its daughter radionuclides, Pb-211 and Bi-211, may accumulate around the patients. These daughter radionuclides of Rn-219 emit alpha particles, which may cause internal exposure of medical staff in the hospital or family of the patients. However, detection of the radon daughters of Rn-219 released from patients during alpha radionuclide therapy has not been reported. The main reason is the difficulty of detection of alpha particles in air. In addition, selectively detecting alpha particles from radon daughters of low-level Rn-219 is extremely difficult or thought to be impossible because natural radon daughters from Rn-222 also exist in the environments^5^. As shown in Fig. 1(B), since natural radon (Rn-222) and her daughter radionuclides (Po-218 and Po-214) emit alpha particles in air, the distinction of radon daughters of Rn-219 from those of Rn-222 is required. The distinction of the alpha-emitting radionuclide from environmental alpha emitters is also required, such as for the detection of plutonium (Pu) in nuclear fuel facilities^6^.

One method is the detection of the difference in daily changes of radon and radon daughter concentrations because natural radon and its daughters from Rn-222 cyclically change with the exhaust system of a room^7^. In this paper, we measured the concentration changes of Rn-222 and daughter radionuclides of Rn-219 and Rn-222 when Ra-223 radionuclide therapy patients were and were not in a room.

Another method is the use of the energy information of the alpha emitters. The energies of the alpha particles emitted from daughter radionuclides of Rn-219 and Rn-222 are different as shown in Table 1. We may be able to distinguish the daughter radionuclides of Rn-219 from those of Rn-222 using the energy information by developing a radon-daughter detector that can also measure the energy of the alpha particles. In this paper, we compared the energy spectra of alpha emitters from daughter radionuclides of Rn-219 and Rn-222 when Ra-223 radionuclide therapy patients were and were not in a room.

**Table 1:** Alpha particles emitted from daughter radionuclides of Rn-219 and Rn-222.

	Rn-219	Rn-222
Half-life	3.94 s	3.82 days
Alpha radionuclides (Energy)	Po-215 (7.4 MeV) Bi-211 (6.6 MeV)	Po-218 (6.0 MeV) Po-214 (7.7 MeV)

We conducted these measurements and confirmed that the concentration of the daughter radionuclides of Rn-219 increased and the energy spectra showed a peak from the daughter radionuclides of Rn-219 when the Ra-223 radionuclide therapy patients were in the room.

## Results

### Measurements of concentrations of Rn-222 and daughter radionuclides of Rn-219 and Rn-222

**Figure 2:**
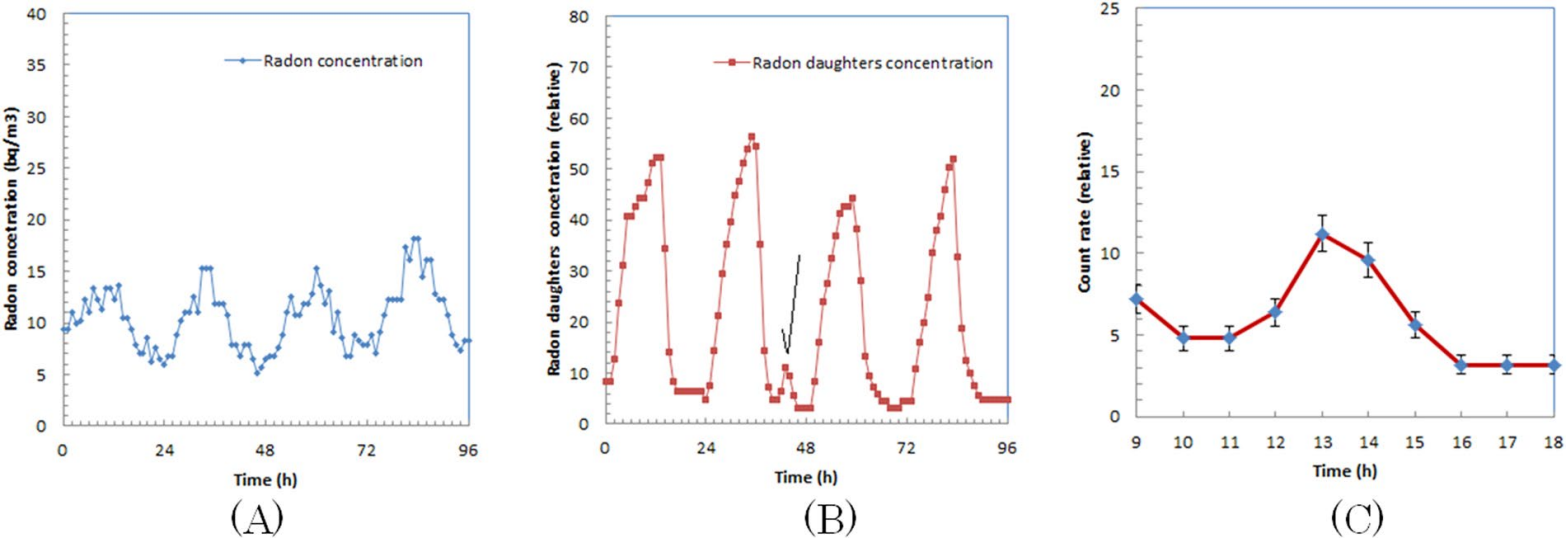
Rn-222 concentration (**A**), concentration and daughter radionuclides of Rn-219 and Rn-222 (**B**), and magnified curve of concentration and daughter radionuclides (**C**) for first trial.

We show the Rn-222 concentration of the room for four days during the first trial of measurements in Fig. 2(A). During the measurement, two patients continuously stayed in the room for 1.5 h. We only observe the daily change of Rn-222 in Fig. 2(A).

The concentration of the daughter radionuclides of Rn-219 and Rn-222 in the same period is shown in Fig. 2(B). We observed an irregular concentration increase in the middle of the horizontal axis as indicated with an arrow. In Fig. 2(C), we show the horizontally expanded concentration change of the daughter radionuclides of Rn-219 and Rn-222 with the periods when the Ra-223-administered patients were in the room. We observed that the concentration increased after the first patient entered the room and peaked when the second patient left the room. The peak concentration of the period was ~3 times higher than the baseline (average of the concentrations at 10 a.m. and 6 p.m.).

**Figure 3:**
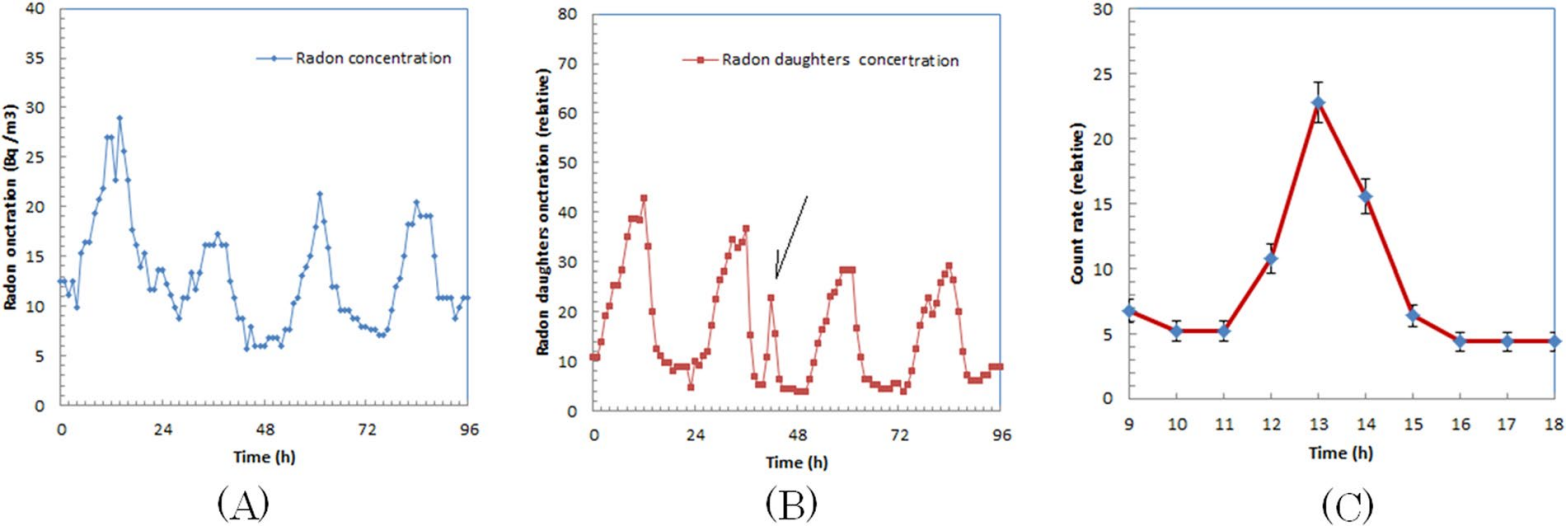
Rn-222 concentration (**A**), concentration and daughter radionuclides of Rn-219 and Rn-222 (**B**), and magnified curve of concentration and daughter radionuclides (**C**) for second trial.

We show the Rn-222 concentration in the room for four days during the second trial of measurements in Fig. 3(A). During the measurement, two patients continuously stayed in the room for 1.5 h. We only observe the daily change of Rn-222 in Fig. 3(A).

The concentration of the daughter radionuclides of Rn-219 and Rn-222 in the same period is shown in Fig. 3(B). We observed an irregular concentration increase in the middle of the horizontal axis as indicated with an arrow. In Fig. 3(C), we show the horizontally expanded concentration change of the daughter radionuclides of Rn-219 and Rn-222 with the periods when the Ra-223-administered patients were in the room. We observed that the concentration increased after the first patient entered the room and peaked when the second patient left the room. The peak concentration of the period was more than 5 times higher than the baseline (average of the concentrations of 10 a.m. and 6 p.m.).

### Measurements of energy spectra of daughter radionuclides of Rn-219 and Rn-222

**Figure 4:**
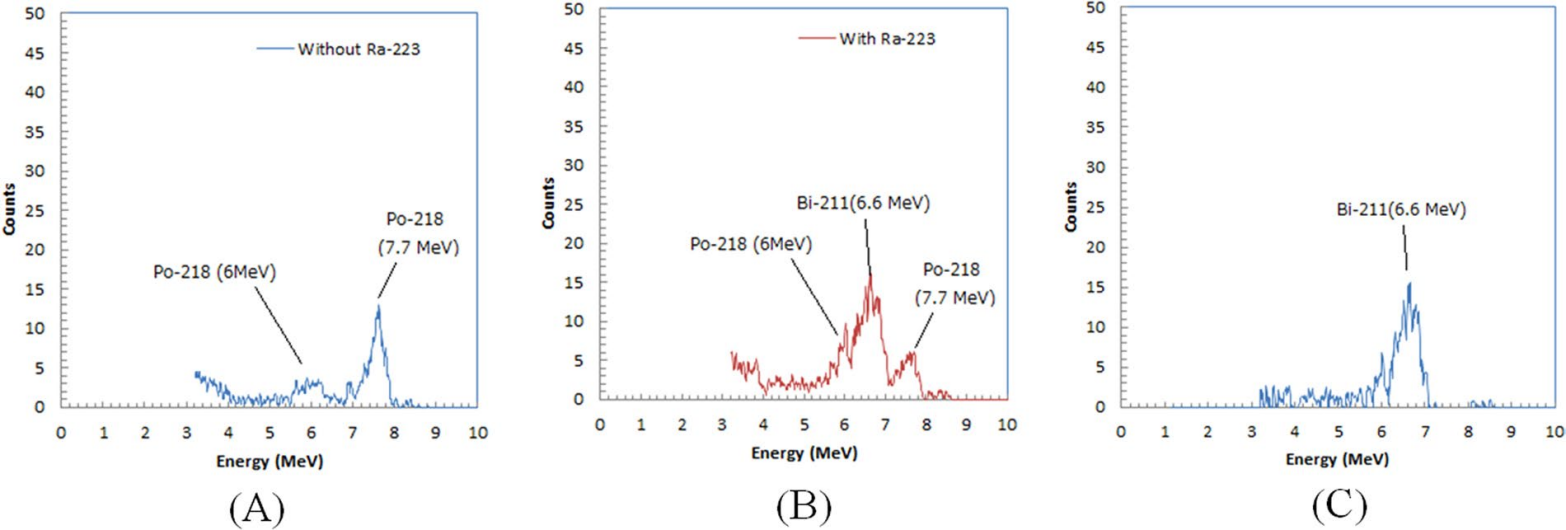
Energy spectra of alpha particles when patients were not in the room (**A**), when those patients were in the room (**B**) and subtracted spectra of these two (**C**).

We show the energy spectra of the alpha particles when the patients were not in the room in Fig. 4(A). The distribution showed peaks for radon daughters of Rn-222, Po-218 (6.0 MeV) and Po-214 (7.7 MeV). We show the energy spectra of the alpha particles when the patients were in the room for the second trial in Fig. 4(B). The distribution showed the highest peak around 6.6 MeV between the peaks of Po-218 (6.0 MeV) and Po-214 (7.7 MeV). The subtracted distribution of these two spectra showed a single peak at 6.6 MeV, which corresponds to the peak from Bi-211 (6.6 MeV), one of the daughter radionuclides of Ra-223 (Fig. 4(C)).

## Discussion

We successfully measured the increases of the daughter-radionuclide concentrations when the Ra-223-administered patients were in the room. We also confirmed that the increases could be attributed to the detection of the alpha particles of Bi-211 (6.6 MeV), one of the daughter radionuclides of Ra-223 emitted from the patients.

The possible alpha particles detected from the daughter radionuclides of Ra-223 were Po-215 (7.4 MeV) and Bi-211 (6.6 MeV). In the energy spectra with the patients shown in Fig. 4(B), we did not observe a significant increase in the energy range for Po-215 (7.4 MeV). This is probably because the half-life of Po-215 is short (1.78 ms), and Po-215 emits alpha particles before it is electrostatically collected to the detector of the radon-daughter detector. However, Po-215 may accumulate around the patients in their mouths or breath.

Although the daughter-radionuclide concentrations when the Ra-223-administered patients were in the room could be detected from the daily change in the concentration and energy spectra, the increase was not very high in our measurement conditions. Although the increased concentration was 4 to 5 times higher than that from the natural alpha emitters, it was lower than the concentration of the natural alpha emitters at night when there was less air ventilation. Thus, the absorbed dose from the alpha particles of daughter radionuclides of Ra-223 will be roughly similar to that of the natural radon and her daughters at ~2 m from the patients. However, closer to the patient, the concentration may increase. In addition, the alpha particles from Po-215 may increase if the distance is very close.

The measurements of the increase in the concentration of the daughter radionuclides of Ra-223 were conducted in the daytime when the air ventilation in the room was higher. If a room has a poor ventilation system, the concentration of the daughter radionuclides of Ra-223 will probably be higher than those shown in Figs 2(C) and 3(C). Thus, a good ventilation system may be important for any room where the patients are administered Ra-223.

## Conclusions

We found that alpha particles of the daughter radionuclides from Ra-223-administered patients were detectable. The concentration was higher than that of the daughter radionuclides from the natural alpha-emitting radioisotope, Rn-222. Thus, it is recommended to conduct proper air ventilation when patients are being treated. However, the increase in levels of alpha emitters is lower than the daily variations and thus is likely not a source of serious concern for radiation exposure.

## Methods

### Detector for measurement of Rn-222 concentration

**Figure 5:**
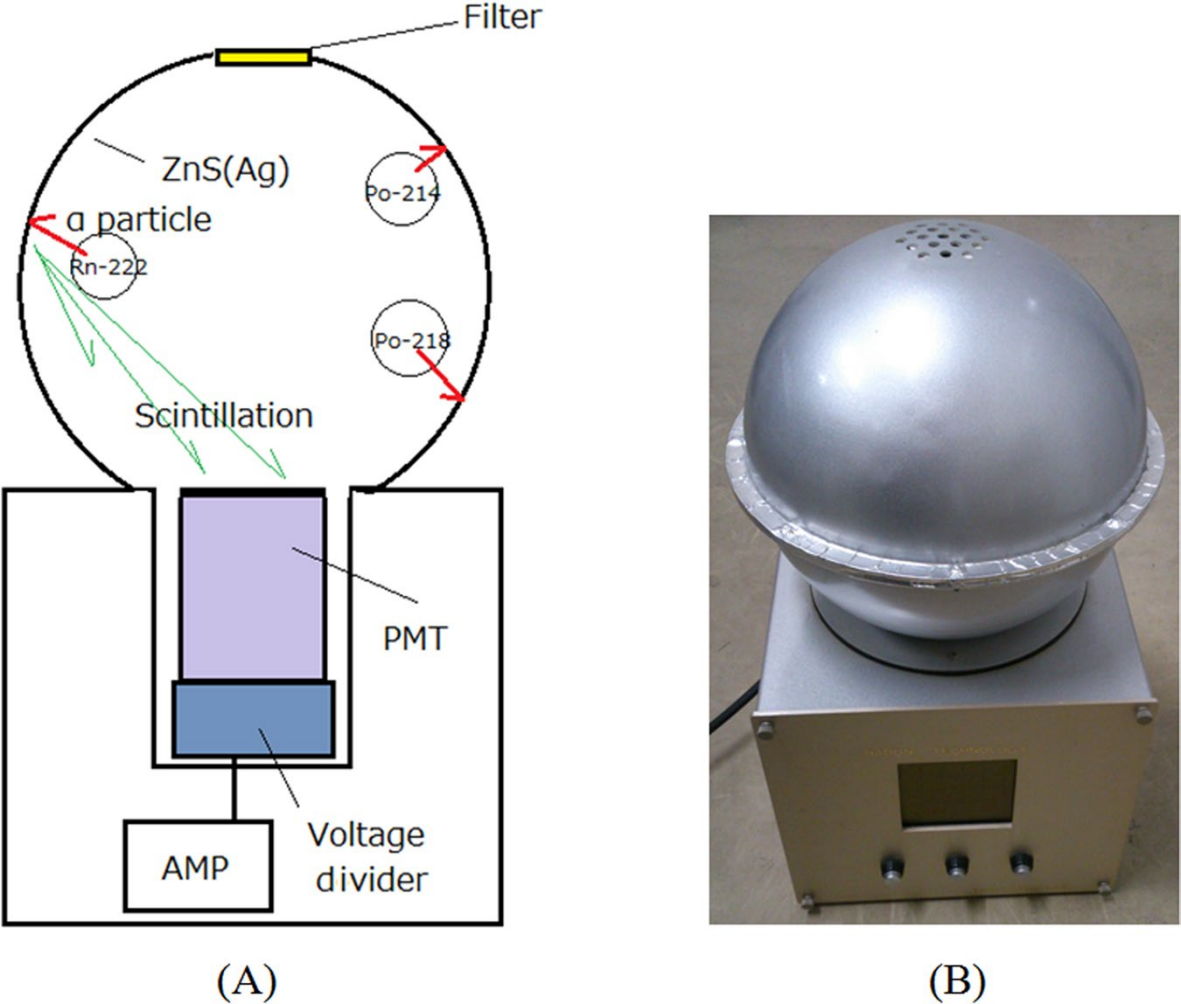
Schematic drawing (**A**) and photo (**B**) of Rn-222 detector.

Measurements of Rn-222 concentration were conducted by a scintillation cell-type detector^8^. A schematic diagram of the developed Rn-222 detector is shown in Fig. 5(A). It was made of a sphere chamber 20 cm in diameter the inside of which was painted with ZnS(Ag) scintillator to detect the alpha particles from Ra-222 and her daughter radionuclides (Po-218 and Po-214). A membrane filter was set at the upper side of the detector to selectively detect Rn-222 concentration. Since the exchange rate of the filter for the inside air was 2 hours, only gas-type radionuclides with a long half-life such as Rn-222 could be detected by the detector^9^. A 2-inch-diameter round photomultiplier tube (PMT) (Hamamatsu R6231) detected the scintillation from the alpha particles from Rn-222 as well as Po-218 and Po-214 decayed inside the chamber. Because the detected alpha particles of Rn-222, Po-218 and Po-214 are proportional to the concentration of Rn-222 inside the chamber, and the concentration of Rn-222 inside the chamber is proportional to the radon concentration in the room, the detector could measure the radon concentration in the room. The output of the PMT was fed to an amplifier and single channel analyzer to measure the count rate change of the Rn-222 concentration in the room. A photo of the developed Rn-222 detector is shown in Fig. 5(B).

### Detector for measurement of daughter radionuclides of Rn-219 and Rn-222

**Figure 6:**
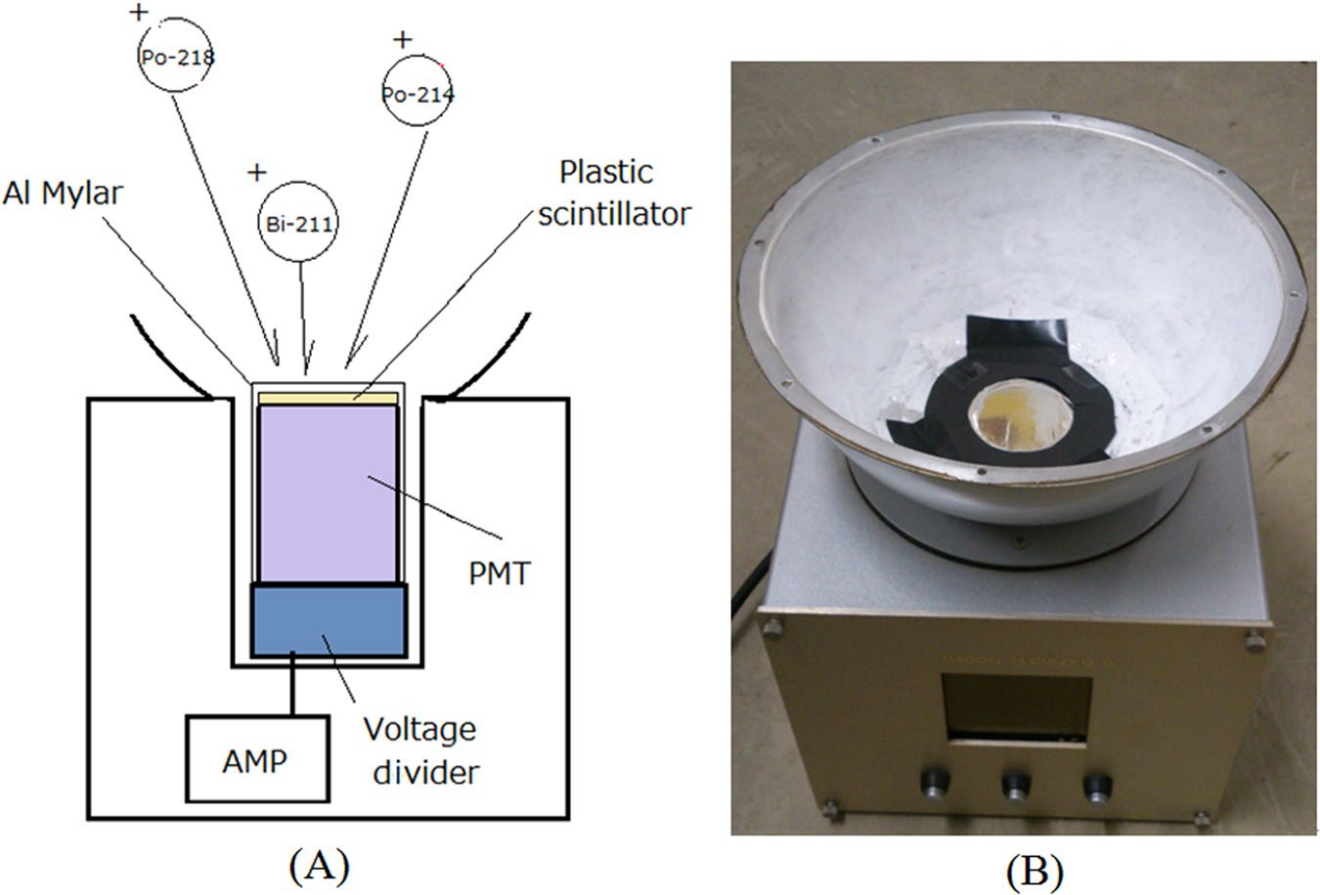
Schematic drawing (**A**) and photo (**B**) of detector for daughter radionuclides of Rn-219 and Rn-222 with energy spectra of alpha particles.

Measurements of daughter radionuclides of Rn-219 and Rn-222 were conducted by an electrostatic collecting-type detector. A schematic diagram of the developed detector is shown in Fig. 6(A). It was made of a scintillation detector using a thin plastic scintillator^10^. Since negative high voltage was supplied to the photocathode of the PMT, the daughter radionuclides of Rn-222 (Po-218 and Po-214) and Rn-219 (Po-215 and Bi-211) accumulated on the plastic scintillator covered with aluminized Mylar because these daughter radionuclides are positively charged by the previous alpha decays^11,12^. The daughter radionuclides of Rn-222 (Po-218 and Po-214) and Rn-219 (Po-215 and Bi-211) emit alpha particles on the plastic scintillator, and the scintillation was detected by a 2-inch-diameter round PMT (Hamamatsu R6231). The output of the PMT was fed to an amplifier and single-channel analyzer to measure the count rate change of the concentrations of the daughter radionuclides of Rn-219 and Rn-222. Because the plastic scintillator is transparent, the scintillation detector can provide energy information. The output of the amplifier was fed to a multichannel analyzer (MCA) (Clear Pulse, 1125P, Japan) to measure the energy spectra of the alpha particles to distinguish daughter radionuclides of Rn-219 from those of Rn-222. Energy spectra measurements were made using the data acquisition software for the MCA (1125 PHA, ver. 1.4.1, Clear Pulse, Japan) and analysis was made with standard Excel software. The calibration of the energy spectra was conducted using an Am-241 alpha source (5.5 MeV) and was confirmed by the peaks of the alpha particles for natural radon daughters, Po-218 (6.0 MeV) and Po-214 (7.7 MeV). A photo of the developed detector for daughter radionuclides of Rn-219 and Rn-222 is shown in Fig. 6(B).

### Measurement of Rn-222 concentration and daughter radionuclides of Rn-219 and Rn-222 with energy spectra

Measurements of Rn-222 concentration and daughter radionuclides of Rn-219 and Rn-222 were conducted in a room located on the first basement floor of the Nagoya University Hospital where a single photon emission tomography system (SPECT) is installed. The ventilation of the room starts at ~8 a.m. and ends at ~6 p.m. every day except on weekends. The concentrations of Rn-222 and daughter radionuclides of Rn-219 and Rn-222 were measured continuously in the room for two months at ~2 m from the bed of the SPECT system where the Ra-223 alpha radionuclide therapy patients were imaged. Four patients in total individually stayed in the room for ~40 min to carry out the SPECT imaging.

For each patient, after the administration of Ra-223 with a radioactivity of ~0.056 MBq/kg (patient weight: 55 kg to 64 kg, age: 69 to 74), the SPECT imaging was conducted from 2 to 3 hours after injection to check the accumulation of Ra-223 in the body. The SPECT imaging was conducted in the room for ~40 min; thus each patient stayed in the room slightly longer than that. The SPECT imaging was conducted for two patients per day continuously from ~11 a.m. to ~1 p.m.

**Figure 7:**
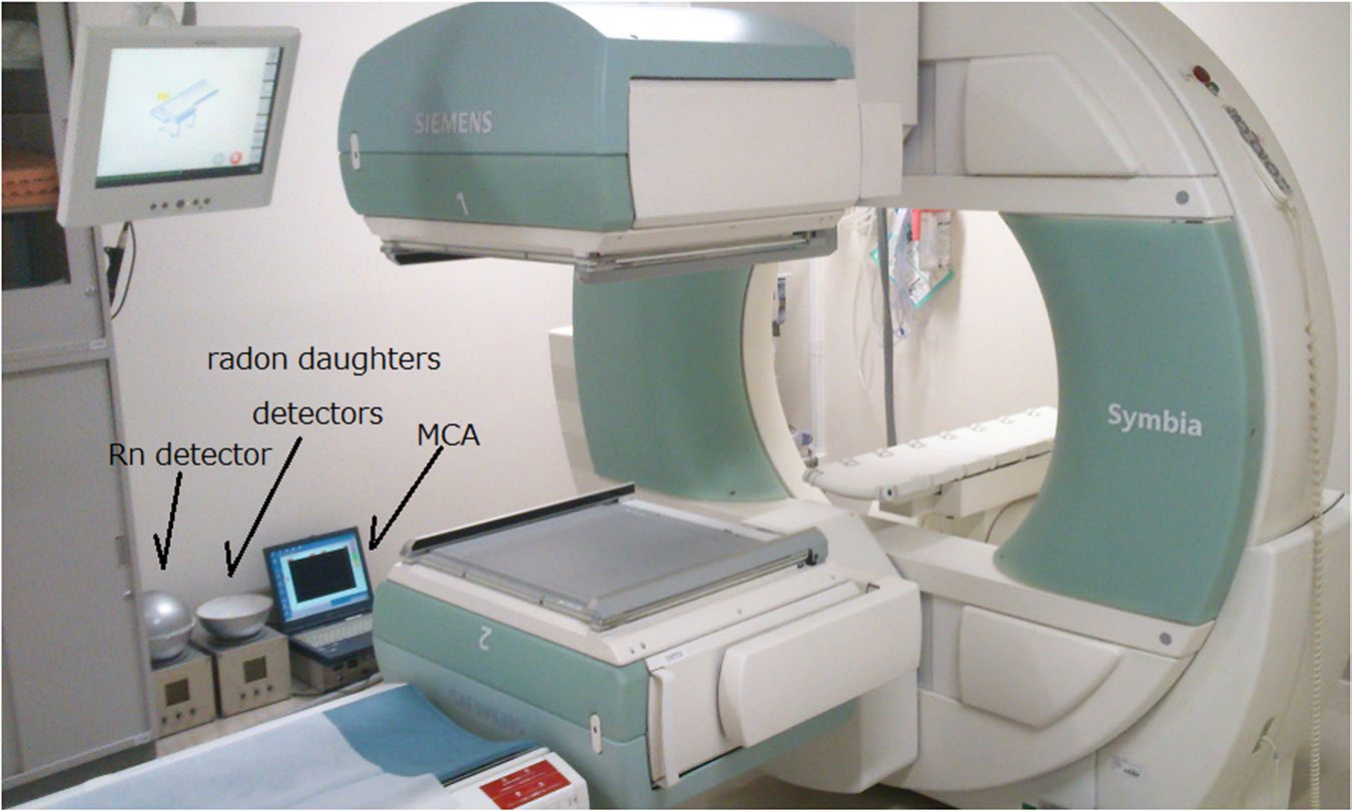
Photo during measurements of Rn-222 concentration and daughter radionuclides of Rn-219 and Rn-222 with energy spectra.

The energy spectra for the daughter radionuclides of Rn-219 were measured on the day of the SPECT imaging of the Ra-223-administered patients. The energy spectra were also measured without patients for comparison. Measurements were conducted for 8 h at the same time of day, 10 a.m. to 6 p.m., to reduce the effect of daily changes in the Rn-222 concentration on the spectra. Figure 7 is a photo taken during measurements of Rn-222 concentration and daughter radionuclides of Rn-219 and Rn-222 with energy spectra in the SPECT room.
